# Stem cell properties of human clonal salivary gland stem cells are enhanced by three-dimensional priming culture in nanofibrous microwells

**DOI:** 10.1186/s13287-018-0829-x

**Published:** 2018-03-22

**Authors:** Hyun-Soo Shin, Songyi Lee, Hye Jin Hong, Young Chang Lim, Won-Gun Koh, Jae-Yol Lim

**Affiliations:** 1 Department of Otorhinolaryngology, Gangnam Severance Hospital, Yonsei University College of Medicine, 211 Eonju-ro, Gangnam-gu, Seoul, 06273 Republic of Korea; 2 Department of Chemical and Biomolecular Engineering, Yonsei University, Seoul, Republic of Korea; 3 Department of Otorhinolaryngology, Head and Neck Surgery, Konkuk University School of Medicine, Seoul, Republic of Korea

**Keywords:** Salivary glands, Xerostomia, Spheroid, Micropatterned nanofibrous scaffolds, Wnt

## Abstract

**Background:**

Three-dimensional (3D) cultures recapitulate the microenvironment of tissue-resident stem cells and enable them to modulate their properties. We determined whether salivary gland-resident stem cells (SGSCs) are primed by a 3D spheroid culture prior to treating irradiation-induced salivary hypofunction using in-vitro coculture and in-vivo transplant models.

**Methods:**

3D spheroid-derived SGSCs (SGSCs^3D^) were obtained from 3D culture in microwells consisting of a nanofiber bottom and cell-repellent hydrogel walls, and were examined for salivary stem or epithelial gene/protein expression, differentiation potential, and paracrine secretory function compared with monolayer-cultured SGSCs (SGSCs^2D^) in vitro and in vivo.

**Results:**

SGSCs^3D^ expressed increased salivary stem cell markers (LGR5 and THY1) and pluripotency markers (POU5F1 and NANOG) compared with SGSCs^2D^. Also, SGSCs^3D^ exhibited enhanced potential to differentiate into salivary epithelial cells upon differentiation induction and increased paracrine secretion as compared to SGSCs^2D^. Wnt signaling was activated by 3D spheroid formation in the microwells and suppression of the Wnt/β-catenin pathway led to reduced stemness of SGSCs^3D^. Enhanced radioprotective properties of SGSCs^3D^ against radiation-induced salivary hypofunction was confirmed by an organotypic 3D coculture and in-vivo transplantation experiments.

**Conclusion:**

The 3D spheroid culture of SGSCs in nanofibrous microwells promotes stem cell properties via activation of Wnt signaling. This may contribute to SGSC priming prior to regenerative therapy to restore salivary hypofunction after radiotherapy.

**Electronic supplementary material:**

The online version of this article (10.1186/s13287-018-0829-x) contains supplementary material, which is available to authorized users.

## Background

Stem cell therapies exploit autologous or allogeneic stem cells or their in-vitro-generated derivatives. Three types of stem cells are generally used to repair and/or replace functionally deteriorated tissues or organs: adult stem cells (ASCs), embryonic stem cells, and induced pluripotent stem cells [1]. Most ASCs are tissue resident and are responsible for maintaining homeostasis or regenerating damaged tissues. ASCs have been transplanted from bone marrow, adipose tissue, peripheral blood, and umbilical cords during cell therapy; however, there are a number of limitations in practice [2, 3]. The cost and labor involved in scale-up and quality control during autologous transplants, and immune reactions following allogeneic transplants, typically limit the clinical use of these cells. Poor engraftment after transplantation irrespective of cell sources is another important issue.

In recent years, an alternative strategy for modulating the stem cell microenvironment or niche has emerged as an attractive method of overcoming the limitations mentioned. The strategy involves stimulating stem cells—including those from transplants or innate ASCs—using exogenous factors, conditioning, or genetic reprogramming [4, 5]. The stem cell niche includes direct interactions between stem cells and neighboring cells, secreted factors, physiological factors, or the extracellular matrix (ECM) [4, 6]. The ECM is a key component of the stem cell niche, and is involved in initiating intracellular signaling and cell fate in nearly all tissues. The interaction between stem cells and the ECM is determined by ECM components and physical properties such as stiffness, elasticity, and topography [4].

Salivary gland-resident stem cells (SGSCs) are salivary gland-resident tissue stem cells which are essential for salivary gland homeostasis and regeneration [7]. We recently isolated single clonal LGR5^+^THY1^+^ SGSCs by subfractionation culture and reported that SGSCs localize in close proximity to the secretory units of the human parotid and submandibular glands in the quiescent state [8]. These SGSCs may be activated when they interact with the basement membrane of the ECM, to which basal cells and myoepithelial cells are anchored. Furthermore, both ourselves and other researchers have recently shown that salivary gland cells are readily assembled into three-dimensional (3D) spheroid-like structures when cultured under 3D culture conditions. Such conditions include embedding in Matrigel, floating in culture, and the use of microcraters or microwell-patterned nanoscaffolds [9–13]. These 3D culture conditions enable the recapitulation of cell–cell and cell–ECM interactions, and have potential for preconditioning or priming stem cells.

In the present study, we employed our microwell culture system, in which SGSCs are self-assembled into 3D spheroids and maintain their stem cell properties without lineage commitment. We determined whether this process influences stem cell function in terms of phenotypic marker expression, differentiation potential, and paracrine activity. We also determined whether 3D spheroid-derived SGSCs (SGSCs^3D^) can remodel radiation damage and restore irradiation (IR)-induced salivary gland hypofunction better than 2D monolayer-cultured SGSCs (SGSCs^2D^) in vitro and in vivo. Our results provide the first proof that 3D priming culture of SGSCs is an alternative prerequisite for modulating stem cell function and overcoming the limitations of cell therapy for salivary gland hypofunction.

## Methods

### Preparation of hydrogel micropatterned nanofibrous microwells

Nanofibrous scaffolds were prepared by a conventional electrospinning process, as described previously [9]. Briefly, an electrospinning solution was prepared by dissolving polycaprolactone (PCL; MW 80,000) in 2,2,2-trifluoroethanol to form a 20% *w*/w solution. The PCL solution was electrospun (NanoNC, Seoul, Korea) for 20 min through a 23-gauge needle with a 6.5-kV positive voltage at a constant flow rate of 0.5 ml/h. The PCL nanofibrous sheets were collected on cover glasses wrapped with clean aluminum foil during the electrospinning process, and the sheets were further treated with oxygen plasma (Femto Science, Kyunggi, Korea) for 2 min to increase the hydrophilicity of the PCL fibers.

The electrospun nanofiber scaffolds were incorporated into hydrogel micropatterns via photolithography, as described in our previous study [9]. First, a hydrogel precursor solution (200 μl) comprising 2-hydroxy-2-methylpropiophenone (2% *v*/v) and poly(ethylene glycol)-diacrylate (50% *w*/w) (PEG-DA; MW 575) in deionized water was dropped onto the electrospun fibers. After the precursor solution had completely spread over the sheet, a photomask representing a square microarray pattern was placed on the nanofiber sheet. Exposure to ultraviolet light (365 nm, EFOS Ultracure 100S S Plus UV spot lamp; EFOS Canada, Mississauga, ON, Canada) for 1 s was sufficient to induce free-radical crosslinking polymerization. After crosslinking, the unreacted precursor solution was washed away with water. For the cell studies, the scaffolds were sterilized in 70% *v*/v ethanol solution for 30 min, and then washed three times in phosphate-buffered saline (PBS) to remove the ethanol. The resultant hydrogel micropattern-incorporated fibrous sheet comprised an array of microwells formed from a nanofiber bottom and a hydrogel wall; we refer to this as a microwell sheet in the present study.

### Cultivation of human single clonal SGSCs

We previously established single SGSC clones isolated from human parotid glands using a modified subfractionation culture method, which has been demonstrated to be effective for the isolation of highly homogeneous clonal stem cells [8]. Briefly, a portion of the normal glands was resected, washed, and chopped with fine scissors and a blade. The minced tissue was then dissociated with 0.05% collagenase II containing Hank’s balanced salt solution. The dissociated tissue solution was then filtered and centrifuged, and the cell pellet was resuspended in Dulbecco’s Modified Eagle’s Medium (Gibco, Grand Island, NY, USA) containing low glucose, 20% fetal bovine serum (Gibco), and 1% penicillin/streptomycin (Gibco). The samples were then incubated in a 100-mm culture dish for 2 h at 37 °C under 5% CO_2_. Next, the cell culture supernatant was transferred to a new 100-mm dish and incubated for 1 h, after which the supernatant was again transferred to a new dish (D1) and incubated for 1 h. The supernatant was subsequently transferred to another new dish (D2) and incubated for 1 day. This process was repeated twice with 1-day incubations (dishes D3 and D4). The single cell-derived colonies that appeared in D2, D3, and D4 were detached with 0.05% trypsin/ethylenediaminetetraacetic acid (EDTA) (Gibco) and isolated using cloning cylinders (Bel-Art Products, Wayne, NJ, USA). They were then transferred to a six-well plate and subsequently to larger culture flasks, where they continued to expand into a number of clonal cell populations. Several single clonal cells were analyzed for their stem cell properties, as described previously [8].

### 2D monolayer and 3D spheroid cultures of SGSCs

We used single cell-derived clones from clonal populations confirmed by examination of stem cell and molecular characteristics. SGSCs at passage 3 were expanded and seeded at a density of 2 × 10^5^ cells/plate; the plates had either been precovered with microwell sheets to enable 3D spheroid formation or were used without a microwell sheet for 2D monolayer culture. To determine the optimum well size, SGSCs were cultured in microwell sheets with 100 × 100 μm^2^, 200 × 200 μm^2^, or 500 × 500 μm^2^ wells for 7 days.

### Cell morphology, viability, and proliferation

Cell morphology was investigated at 1, 3, 5, and 7 days after culture using an inverted phase-contrast microscope (Olympus FV1000; Olympus, Tokyo, Japan), and images were obtained using a digital camera. To assess cell viability, a LIVE/DEAD assay kit (Invitrogen, Carlsbad, CA, USA) was utilized according to the manufacturer’s instructions. Briefly, SGSCs were seeded into microwell sheet-covered six-well plates at a density of 2 × 10^5^ cells/plate. On the fifth day of culture, cell viability was measured by exposing the cells to the LIVE/DEAD reagent (2 mM ethidium homodimer-1 (EthD)-green for live cells; 4 mM Calcein AM-red for dead cells) for 30 min at 25 °C. The cells were then visualized under an Axiovert 200 fluorescence microscope (Carl Zeiss, Oberkochen, Germany).

We conducted a 3-(4,5-dimethylthiazol-2-yl)-5-(3-carboxymethoxyphenyl)-2-(4-sulfophenyl)-2H-tetrazolium (MTS) uptake assay to assess the proliferation of the SGSCs. Briefly, the SGSCs were cultured on substrates in a 96-well plate at a density of 1 × 10^4^ cells/well, and cell proliferation was observed at 1, 3, and 5 days after culture. After adding 20 μl of MTS reagent and incubating at 37 °C for 4 h, the proliferation of the SGSCs was determined by measuring the absorbance at 490 nm in triplicate using a 96-well plate reader (Dynex Revelation; Dynex Ltd, Billingshurst, UK). At least three independent cell viability and proliferation analyses were performed.

In addition, we performed a Trypan blue dye exclusion assay, in which intact cell membranes exclude the dye. On the fifth day, the cells were washed once with 1× PBS, detached using 0.05% Trypsin–EDTA, neutralized with cell culture media, and collected in 50-ml tubes. The cells were centrifuged at 127 × *g* for 5 min, then the supernatant was discarded and the pellets were resuspended in Trypan blue dye in media for 10 min before cell counting using a hemocytometer. The cell viability percentage was determined based on the viable cell count divided by the total cell count.

### Evaluation of phenotypic gene and protein expression

#### Flow cytometry

The 3D spheroid-derived SGSCs (SGSCs^3D^) were subjected to flow cytometry to investigate cell surface marker proteins. Briefly, the cells were washed twice with PBS, harvested by treatment with trypsin/EDTA, and incubated with fluorescein isothiocyanate (FITC) or phycoerythrin (PE)-conjugated antibodies. The cells were then investigated using a FACSCalibur system (BD Biosciences, Franklin Lakes, NJ, USA), after which the data were analyzed using CellQuest software (BD Biosciences, San Jose, CA, USA). The following antibodies were used for flow cytometric analysis: CD29 (BD Biosciences), CD73 (BD Biosciences), CD90 (THY1; R&D Systems, Minneapolis, MN, USA), CD105 (BD Biosciences), and LGR5 (Thermo Fisher Scientific) for salivary stem cell markers; CD45 (BD Biosciences) and HLA-DR (R&D Systems) for hematopoietic markers; and OCT4 (R&D Systems) for embryonic markers. Isotype-matched control antibodies were used in each antibody analysis. At least three independent experiments were performed.

#### Quantitative real-time polymerase chain reaction analysis

The levels of transcripts of SGSCs^2D^ and SGSCs^3D^ were determined by real-time polymerase chain reaction (PCR) using an ABI PRISM sequence detection system with SYBR Green I as a double-stranded DNA-specific dye according to the manufacturer’s instructions (Applied Biosystems, Foster City, CA, USA). The PCR was carried out using 1 μM complementary DNA (cDNA), 10 μM SYBR Green PCR master mix (Roche Diagnostics, Basel, Switzerland), and 10 pM sense and antisense primers specific for each gene (Additional file 1: Table S1). The relative expression levels were determined by real-time PCR in three independent experiments conducted in triplicate for each sample, and the results were normalized to the housekeeping gene *GAPDH*.

#### Western blotting analysis

Samples of SGSCs^2D^ and SGSCs^3D^ were isolated from the lysate (30 μg), mixed in reducing buffer, boiled, resolved on sodium dodecyl sulfate polyacrylamide gel electrophoresis (SDS-PAGE) gels, and transferred to a polyvinylidene fluoride membrane by blotting. The blot was incubated overnight at 4 °C in blocking solution with primary antibodies to the following antigens: α-amylase, AQP5, TJP1, β-catenin, lamin-B1, and β-actin (Santa Cruz Biotechnology, Santa Cruz, CA, USA); E-cadherin (BD Biosciences); KRT5, NANOG, POU5F1, and SOX2 (Abcam, Cambridge, UK); WNT3A, AXIN2, and p-GSK3β (Cell Signaling Technology, Danvers, MA, USA); and LGR5 (Thermo Scientific, Waltham, MA, USA). The blots were washed with 0.1% Tween 20 in 1× PBS, incubated with horseradish peroxidase-conjugated secondary antibodies corresponding to each primary antibody, and subjected to enhanced chemiluminescence detection (GE Healthcare Life Science, Piscataway, NJ, USA). The protein band intensities were quantified in three independent experiments, and the relative ratios were calculated using β-actin as a reference.

#### Immunofluorescence analysis

For immunofluorescence staining, SGSCs^2D^ and SGSCs^3D^ were seeded on substrates in six-well plates at a density of 2 × 10^5^ cells/well. On the fifth day of culture, they were washed and fixed in 4% paraformaldehyde (20 min at 25 °C), then permeabilized with 0.4% Triton X-100 in 1× PBS (10 min at 25 °C). The cells were then treated with 1% bovine serum albumin (BSA) in 1× PBS for 1 h before incubation in 1% BSA overnight at 4 °C with primary antibodies specific for: α-amylase and TJP1 (Santa Cruz Biotechnology), Occludin (Thermo Scientific), AQP5 (Alomone Lab, Jerusalem, Israel), E-cadherin (BD Biosciences), F-actin and THY1 (Abcam), and LGR5 (Thermo Scientific). The cells were then washed in PBS before incubation with goat anti-mouse IgG-Alexa-488-conjugated and goat anti-rabbit IgG-Alexa-555 secondary antibodies (Invitrogen) for 6 h at 25 °C in the dark. The nuclei were counterstained with 4′,6-diamidino-2-phenylindole dihydrochloride (DAPI; Vector Labs, Burlingame, CA, USA), and the cells were examined using a confocal laser scanning microscope (Olympus FV1000).

#### Evaluation of epithelial structural integrity

Confocal microscope images were acquired at magnifications of 20× (dry objective) and 40× (oil immersion objective). XZ slices (0.8-μm thick) for 20× magnification were acquired for samples. Quantitative images were captured at the same depth using the same laser intensity and gain settings so that fluorescence intensities could be compared based on cross-sectional views of samples. To assess cell polarity, projection images of the Z-stack were analyzed using ImageJ software (NIH, Bethesda, MD, USA). Specifically, images were acquired from cross-sectional views of Z-stacks, and 3D reconstructions of each sample were generated using the RGB Plot plugin.

### Evaluation of differentiation potentials

After 5 days of culture in microwell sheets or on monolayer cultures, the cultures were washed with 1× PBS, detached using 0.05% Trypsin–EDTA, and neutralized with cell culture media. The dissociated cells were counted and SGSCs^2D^ or SGSCs^3D^ were plated at a density of 2 × 10^5^ cells/plate; each plate had six wells and had been precovered with Matrigel. The medium was changed to hepato-STIM medium (BD Biosciences) supplemented with recombinant epidermal growth factor (EGF; BD Biosciences), 2 mM l-glutamine, and 1% penicillin/streptomycin, and the cells were incubated at 37 °C for 5 days. To evaluate the differentiation potential, organoid-forming efficacy on Matrigel was determined by measuring the average number of organoids per plates after inducing differentiation from the same number of SGSCs^2D^ or SGSCs^3D^.

### Investigation of mechanism of promotion of stem cell properties

#### Microarray

Total RNA was isolated using the TRIzol Reagent (Molecular Research Center) and delivered to eBiogen for microarray procedures. The complementary RNA was applied to the Agilent human and mouse GE 4 × 44 K v2 Microarray (Agilent Technologies, Santa Clara, CA, USA). Differentially expressed genes (DEGs; fold-changes > 2 and *P* < 0.05) were analyzed using the DAVID bioinformatics tool (v6.7; NIAID/NIH). The functional annotation of genes was performed using the Gene Ontology Consortium database (http://www.geneontology.org). Pathway analysis was carried out using the KEGG pathway database.

#### Transfection of small interfering RNA or plasmids

To determine the molecular mechanisms associated with the enhancement of stemness by 3D spheroid culture, we investigated the effects of *WNT3A* and *β-catenin* gene silencing by transfection with small interfering RNA (siRNA) against human WNT3A and β-catenin (Thermo Scientific). For *WNT3A and β-catenin* gene silencing, siRNA transfection was conducted using Lipofectamine RNAiMAX™ (Invitrogen) with the following siRNAs: WNT3A (100 pM, Accell SMARTpool human WNT3A siRNA) and β-catenin (100 pM, Accell SMARTpool human β-catenin siRNA). Scrambled siRNA from a nontargeting siRNA pool (Thermo Scientific) served as a control.

For overexpression by transfection with a β-catenin plasmid, SGSCs were seeded into six-well plates and incubated for 24 h until 80% confluence was reached, followed by transfection of a control pcDNA3-HA plasmid (1 μg) or a pcDNA-HA β-catenin plasmid (1 μg) using Lipofectamine 2000 (Invitrogen) according to the manufacturer’s protocol. After 48 h, the cells were harvested and the protein was isolated.

#### WNT3A and R-spondin treatment and inhibition of WNT/β-catenin signaling

To evaluate the function of WNT signaling, a WNT agonist (100 ng/ml; R&D Systems) and R-spondin (0.25 μg/ml; R&D Systems) were added to the 2D monolayer and 3D spheroid culture. WNT3A and R-spondin were preincubated with the cells before culture.

### 3D organotypic coculture experiment

We isolated and cultured human parotid epithelial cells (hPECs), as described previously [9]. Specimens were collected with informed consent and institutional review board approval. The hPECs were seeded in the Matrigel-precoated lower chamber at a density of 10^5^ cells/well, and allowed to aggregate to form 3D spheroids on GFR-Matrigel for 3 days. The cells were then irradiated at 10 Gy, which corresponds to the half-maximal inhibitory concentration (IC_50_) according to our previous study using 4-MV X-rays from a linear accelerator (Mevatron MD) [14]. For the coculture experiment, irradiated 3D hPEC spheroids were cocultured with either SGSCs^2D^ or SGSCs^3D^ cultured in the upper Transwell inserts at 37 °C for 48 h. Cellular, structural, and functional changes in IR-affected hPEC spheroids were then evaluated after coculture with SGSCs^2D^ or SGSCs^3D^, as described previously [14]. To explore the therapeutic effects of factors secreted from the hSGSCs into the medium, a coculture experiment was conducted under serum-free conditions in keratinocyte serum-free medium.

### Functional analyses of 3D assembled structures

#### Amylase activity

To measure the secretory function of hPECs, the activity of amylase released from hPECs in 3D spheroids was evaluated. hPECs were seeded on Matrigel at 2 × 10^5^ cells per well in six-well plates, cultured for 3 days, and then irradiated at 10 Gy. The hPECs in 3D spheroids were stimulated with epinephrine (Epi) 10 μM or isoproterenol (ISO) 1 μM in culture medium for 45 min before measurement. Amylase activity was measured from medium collected at 2 days after IR using an α-amylase activity assay kit (Abcam, Cambridge, MA, USA) with 2-chloro-*p*-nitrophenol linked to maltotriose as the chromogenic substrate according to the manufacturer’s instructions. The α-amylase activity of the sample was directly proportional to the increase in absorbance at 405 nm observed using a standard laboratory plate reader. The absorbance (OD) was measured in triplicate from three independent experiments and data were normalized to the total number of counted cells. The culture medium without cells was considered a negative control.

##### Measurement of intracellular CaCl_2_

Intracellular calcium ([Ca^2+^]_*i*_) in the 3D spheroids was measured under the same conditions using the calcium-sensitive fluorescence indicator Fluo-4 AM (Molecular Probes, Carlsbad, CA, USA), according to the manufacturer’s instructions. The acinar-like 3D spheroids were incubated with Fluo-4 AM 10 μM in KSFM for 1 h at 37 °C, after which they were washed twice with PBS and measurements were accomplished without calcium. To stimulate Ca^2+^ influx, Carbachol 10 μM (Sigma, St. Louis, MO, USA), adenosine triphosphate (ATP) 100 μM, or thapsigargin 1 μM was added to the medium. The Fluo-4 AM signal was then recorded using live cell imaging and confocal microscopy (Olympus FV1000), after which the fluorescence intensity at the baseline and post stimulation were measured from three independent experiments and compared among groups.

### Transplantation of 3D-cultured SGSCs

Twenty-four 6-week old female C3H mice weighing 18–20 g were purchased from the Research Model Producing Center (Orient Bio Inc., Gyeonggi-do, Korea). Procedures and maintenance were performed in accordance with the Institutional Guidelines and Use Committee approved by the Institutional Animal Ethics Committee (Permit Number 150716–371). The mice were divided randomly into four groups as follows: group 1, Sham (*n* = 6); group 2, 15 Gy IR + vehicle (PBS) injection (*n* = 6); group 3, 15 Gy IR + SGSC^2D^ injection (*n* = 6); and group 4, 15 Gy IR + SGSC^3D^ injection (*n* = 6).

To induce IR-induced SG hypofunction, the mice were anesthetized by intraperitoneal injection with xylazine (10 mg/kg) and ketamine (110 mg/kg). Mice from groups 2, 3, and 4 were firmly held on a monolayer mold, and their necks, including their salivary glands, were irradiated. Irradiation was conducted using a 4-MV X-ray linear accelerator (Mevatron MD) at a single dose of 15 Gy, with a focus-to-skin distance of 100 cm. The mice in the sham group did not receive IR.

After 4 weeks of IR, the mice were anesthetized and a 5-mm incision in the neck was made to expose the submandibular gland. Either SGSCs^2D^ or SGSCs^3D^ were injected (2 × 10^5^ cells in 10 μl of PBS) into both submandibular glands of the mice in groups 3 and 4 using a Hamilton syringe. The same volume of PBS was injected into the glands of the group 2 mice, after which the neck wound was sutured and sterilized. Stem cell engraftment, macromorphological and micromorphological changes, and salivary functions were evaluated, as described previously [8].

### Statistical analysis

Statistical analysis was conducted using the GraphPad Prism 5 package (GraphPad Software, La Jolla, CA, USA). The Mann–Whitney test was used to assess differences between groups, and one-way analysis of variance (ANOVA) followed by Tukey’s post hoc test and two-way ANOVA followed by the Bonferroni post hoc test were used to analyze differences among groups. Linear regression was applied to evaluate the correlation between parameters. *P* < 0.05 was considered to indicate statistical significance.

## Results

### Single clonal SGSCs retain multipotent tissue stem cell characteristics

The culture-expanded SGSCs maintained fibroblast-like morphological consistency and constantly proliferated during subculture. To measure the CFU efficiency, SGSCs at passage 3 were seeded at 100 cells/well in a six-well plate and cultured for 10 days, after which the cells were stained with crystal violet and all colonies positive for crystal violet were counted. The CFU activity (average number of colonies formed/total number of seeded cells × 100) was calculated. As shown in Additional file 2: Figure S1a, the CFU efficiency of the SGSCs was 25.7% ± 1.3%. Next, we examined the surface marker expression by flow cytometric analysis. The SGSC clones were positive for mesenchymal (CD29, CD73, CD90, CD105), epithelial (LGR5), and pluripotent (OCT4) stem cell markers, but negative for hematopoietic stem cell markers (CD45 and HLA-DR) (Additional file 2: Figure S1b). We then explored whether the SGSC clones possessed multipotency to give rise to mesenchymal or epithelial lineages. Upon the induction of mesenchymal differentiation, clonal SGSCs exhibited fat, bone, and cartilage phenotypes (Additional file 2: Figure S1c). The expression of lineage-specific molecular markers (adipogenic markers (*PPARγ2, FABP4,* and *LPL*), osteogenic markers (*RUNX2* and *BGLAP;* osteocalcin), and chondrogenic markers (*COL2,* type II collagen; *COL10,* type X collagen; and *ACAN,* aggrecan)) was also confirmed (Additional file 2: Figure S1c).

Furthermore, SGSC clones were capable of differentiation into hepatocytes upon hepatogenic induction (Additional file 2: Figure S1d). When the SGSC clones were seeded onto Matrigel-coated wells and cultured in differentiation medium, the cells formed spherical cell aggregates that significantly expressed the salivary acinar markers AQP-5 and α-amylase (Additional file 2: Figure S1e). Collectively, these results indicated that culture-expanded single clonal SGSCs were compatible with multipotent tissue stem cell clones in SGs.

### Determination of optimal 3D spheroid culture conditions in microwells

We next determined the effects of 2D monolayer and 3D microwell cultures on viability and the proliferation of SGSCs using LIVE/DEAD, Trypan blue, and MTS assays. Although a few dead cells (red fluorescence) were observed in the middle of the spheroids during culture, there were no significant differences in cell death between the groups with regard to the LIVE/DEAD assay (Additional file 2: Figure S2a). Furthermore, the percent cell viability in the Trypan blue exclusion assay, in which dead cells were counted to measure the percentage of live cells, was preserved in each group during induction of spheroid formation (Additional file 2: Figure S2b). The MTS assay revealed that SGSC proliferation was greater in the microwells than in the 2D monolayer culture (Additional file 2: Figure S2c). These findings indicate that 3D spheroid culture in microwells exerted no significant cytotoxicity and maintained cell proliferation without significant cell death.

To determine the optimal size (length along each *x* and *y* axis) of the wells, SGSCs were cultured in microwell sheets with well sizes of 100, 200, and 500 μm^2^. The SGSCs were cultured for 5 days, after which morphological changes and spheroid organization were compared with those of SGSCs cultured on a 2D monolayer (SGSCs^2D^) as a control. The results showed that 3D culture in microwells resulted in morphological changes in which SGSCs aggregated to form spheroid structures over time (Additional file 2: Figure S2d). The 3D spheroid assembly of SGSCs was greatest with a well diameter of 200 μm, in which SGSCs were structurally well organized into 3D spheroids, whereas the SGSCs did not form organized spheroids in 100 and 500 μm^2^ wells (Additional file 2: Figure S2d). Furthermore, dissociated SGSCs from 3D spheroids (SGSCs^3D^) in 200 μm^2^ wells expressed more stem cell-related markers, including pluripotent stem cell markers (*POU5F1* and *NANOG*) and salivary stem/progenitor cell markers (*LGR5* and *THY1*), than those in 100 or 500 μm^2^ wells (Additional file 2: Figure S2e).

### 3D-assembled SGSC spheroids have enhanced stem cell properties

#### Enhanced differentiation potential

**Fig. 1:**
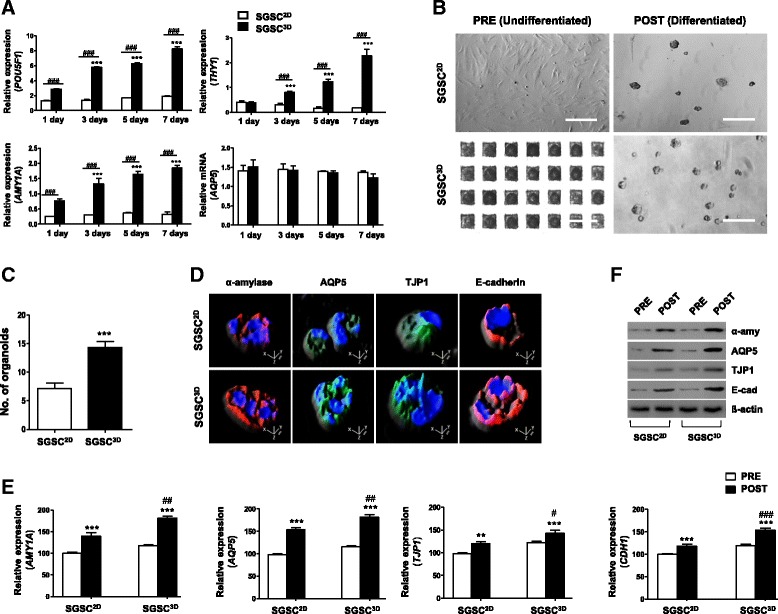
Enhanced differentiation potential of 3D-assembled SGSC spheroids. **a** Transcription levels of salivary acinar (*AMY1A* and *AQP5*) and stem cell-related (*POU5F1* and *THY1*) markers compared by qPCR at 1, 3, 5, and 7 days between 2D monolayer-cultured SGSCs (SGSCs^2D^) and 3D spheroid-derived SGSCs (SGSCs^3D^) culture. Data from three independent experiments analyzed and presented as mean ± SEM (*n* = 3). Two-way ANOVA, Bonferroni’s post hoc test. *Compared with Day 1; ^#^compared with SGSCs^2D^ in each group. **b** Light microscope images of SGSCs after differentiation. Scale bars represent 400 μm. **c** Differentiation capacity determined by measuring average number of acinus-like organoids per plate after plating same number of cells. Data from five independent experiments analyzed and presented as mean ± SEM (*n* = 5). One-way ANOVA; Tukey’s post hoc test. *Compared with monolayer-cultured salivary gland-resident stem cells (SGSCs^2D^). **d** Immunofluorescent images representing salivary acinar markers α-amylase (red) and AQP5 (green), tight junction protein TJP1 (green), and adherence protein E-cadherin (red). Scale bars represent 20 μm. **e** mRNA levels of SG acinar cell markers (A*MY1A* and *AQP5*), tight junction gene (*TJP1*), and intercellular adherence gene (*CDH1*) determined by real-time PCR in SGSC^2D^ and SGSC^3D^ cultures after differentiation. All qPCR measurements performed in triplicate. **f** Protein levels of α-amylase, AQP5, TJP1, and E-cadherin determined by western blotting in SGSC^2D^ and SGSC^3D^ cultures after differentiation. **P < 0.01, ***P < 0.001, ^#^*P* < 0.05, ^##^*P* < 0.01, ^###^*P* < 0.001. SGSC salivary gland-resident stem cell

Next, to determine whether the SGSCs maintained stem cell properties and were not induced to differentiate in the nanofibrous microwells, changes in the mRNA transcripts from SGSCs^3D^ including salivary acinar markers (*AMY1A* and *AQP5*) and stem cell-related markers (*POU5F1* and *THY1*) were evaluated by quantitative-PCR at 1, 3, 5, and 7 days after culture (Fig. 1a). mRNA expression related to stem cell markers increased in SGSCs^3D^ over time, but salivary epithelial transcripts did not increase for 7 days (Fig. 1a), suggesting that stem cell properties were promoted without salivary acinar cell commitment using 3D spheroid culture in microwells, and that spheroid-derived SGSCs (SGSCs^3D^) maintained their enhanced stemness.

**Fig. 2:**
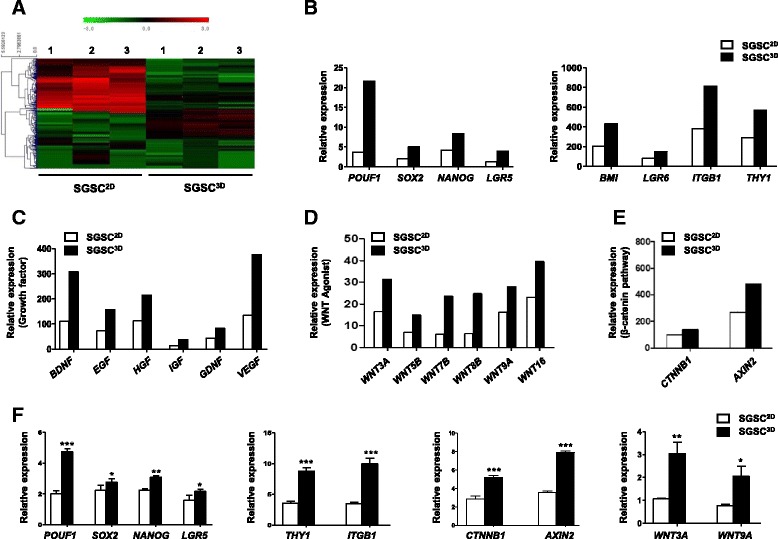
Effects of 3D priming culture on stemness and paracrine secretion. **a** Heat map showing differential expression of genes in microarrays of monolayer-cultured SGSCs (SGSCs^2D^) and 3D spheroid-derived SGSCs (SGSCs^3D^). DEGs (> 2-fold) quantified and antibody reactivity intensity indicated as red pixels (values > 2-fold) and green pixels (values < 2-fold). Stem cell-related genes (**b**), growth factor genes (**c**), and WNT-β-catenin-related genes (**d**, **e**) in SGSCs^3D^ after whole-genome microarray analysis compared with SGSCs^2D^. **f** Confirmation of microarray results for stem cell markers and WNT-β-catenin-related markers after real-time PCR. Data from three independent experiments analyzed and presented as mean ± SEM (*n* = 3). One-way ANOVA; Tukey’s post hoc test. *Compared with SGSCs^2D^. *P < 0.05, **P < 0.01, ***P < 0.001. *SGSC* salivary gland-resident stem cell, *EGF* epidermal growth factor, *HGF* hepatocyte growth factor, *IGF* insulin growth factor, *VEGF* vascular endothelial growth factor, *BDNF* brain-derived neurotrophic factor, *GDNF* glial cell line-derived neurotrophic factor

We next determined whether SGSCs can give rise to salivary epithelial cells upon induction to evaluate the differentiation potential of the SGSCs. The same number of SGSCs^3D^ and SGSCs^2D^ were seeded onto Matrigel-coated plates, with a medium change to serum-free hepato-STIM medium, in which the cells aggregated to form acinar-like organoid structures during 5 days of induction (Fig. 1b). SGSCs^3D^ produced acinar-like structures more efficiently than the SGSC^2D^ control. The differentiation capacity was confirmed by measuring the numbers of acinar-like organoids at 5 days after plating the same number of SGSCs^2D^ and SGSCs^3D^, and SGSCs^3D^ formed more organoids compared with SGSCs^2D^ (Fig. 1b, c). Specifically, the SGSC^3D^ spheroids expressed higher levels of the acinar markers α-amylase (a functional salivary protein) and AQP5 (a water channel protein), tight junction protein (TJP1, ZO-1), and E-cadherin (Fig. 1d). To evaluate the 3D distribution of salivary phenotypic markers, we analyzed a confocal Z-stack with XZ slices. Reconstruction of projection images of the Z-stack into 3D images revealed that acinar-like organoids were more spherically organized from SGSCs^3D^ than SGSCs^2D^ (Fig. 1d). The corresponding genes and proteins were also highly expressed in SGSCs^3D^ after differentiation induction (Figs. 1e and 2f, densitometry shown in Additional file 2: Figure S3). These results suggest that SGSCs can differentiate and assemble acinar-like organoid structures on Matrigel in induction medium and the differentiation potential increased when the cells were primed under 3D culture in microwells compared with those in 2D monolayer culture.

#### Microarray data

We next conducted microarray analysis to compare gene expression between SGSCs^2D^ and SGSCs^3D^ obtained from three donors (Fig. 2a). Differentially expressed gene (DEG) analysis indicated that the expression of stem cell-related genes (*POU5F1*, *SOX2*, *NANOG*, *BMI1*, *LGR5*, *LGR6*, *ITGB1*, and *THY1*) was enriched in SGSCs^3D^ relative to expression of such genes in SGSCs^2D^, indicating enhancement of stemness after 3D spheroid culture in microwells (Fig. 2b). Paracrine activity was also enhanced, and SGSCs^3D^ expressed more antiapoptotic or angiogenic growth factor genes than the SGSC^2D^ control (Fig. 2c). The concentration of growth factors was confirmed by ELISAs of the culture medium of SGSCs^2D^ and SGSCs^3D^. The results showed that 3D culture resulted in a significant increase in EGF, HGF, IGF, and VEGF production relative to monolayer culture of SGSCs (Additional file 2: Figure S4). Greater numbers of WNTs (*WNT3A*, *WNT5B*, *WNT7B*, *WNT8B*, *WNT9A*, and *WNT16*) and their target signaling genes (*CTNNB1* and *AXIN2*) were upregulated in SGSCs^3D^ compared with in SGSCs^2D^ (Fig. 2d, e). Changes in stem cell-related genes and WNT/β-catenin signaling genes were confirmed by quantitative PCR (Fig. 2f).

### Stem cell properties are enhanced by WNT activation following priming in 3D microwell culture

**Fig. 3:**
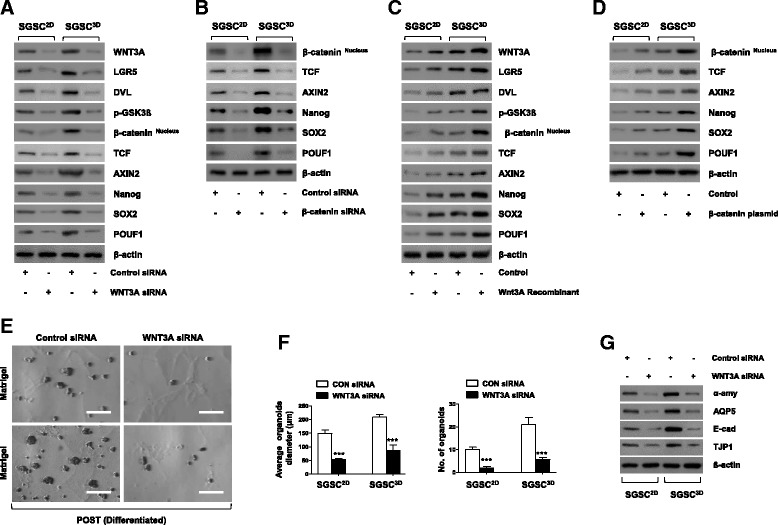
WNT activation and signaling following priming in 3D microwell culture. **a**, **b** Presence of WNT3A and β-catenin siRNA suppressed expression of WNT-β-catenin-related and stem cell-related proteins. **c**, **d** Recombinant WNT3A and β-catenin plasmid upregulated WNT3A-β-catenin-related and stem cell-related proteins. **e** Knockdown of WNT3A reduced differentiation capacity of SGSCs and decreased formation of acinus-like organoids. Scale bars represent 100 μm. **f** Diameter of acinus-like organoids normalized to total number of spheroids measured after knockdown of WNT3A. Differentiation capacity also determined by measuring average number of organoids per plate after plating same number of cells. Data from five independent experiments analyzed and presented as mean ± SEM (*n* = 5). One-way ANOVA; Tukey’s post hoc test. *Compared with control siRNA. ***P < 0.001. **g** Protein levels of SG acinar cell markers (α-amylase and AQP5), tight junction proteins (TJP1), and intercellular adherence protein (E-cadherin) determined by western blotting in acinus-like organoids after induction of differentiation in presence of WNT3A siRNA. SGSC salivary gland-resident stem cell, SGSCs^2D^ monolayer-cultured SGSCs, SGSCs^3D^ 3D spheroid-derived SGSCs, siRNA small interfering RNA, CON control

To confirm whether Wnt/β-catenin signals enhanced the stemness of SGSCs, we investigated the effects of WNT activation, knockdown, or knockin on changes in the Wnt/β-catenin signaling pathway and the stem cell functions of SGSCs. The expression of WNT3A, LGR5, DVL, phosphorylation-GSK3β, nuclear β-catenin, TCF, and AXIN2 increased in SGSCs^3D^ in the microwells (Additional file 2: Figure S5a). However, in the presence of WNT3A or β-catenin siRNAs, the expression of downstream signal and target signal proteins including NANOG, SOX2, and POU5F1 was downregulated (Fig. 3a, b). We also observed that Wnt agonists including WNT3A and R-spondin activated the Wnt/β-catenin pathway and induced the expression of stem cell markers such as NANOG, SOX2, and POU5F1 (Fig. 3c and Additional file 2: Figure S5b). Furthermore, overexpression of the β-catenin in the SGSCs by knockin increased the expression of downstream target signals (TCF, AXIN2, NANOG, SOX2, and POU5F1) (Fig. 3d).

Next, we determined whether WNT activation promotes stem cell function in terms of differentiation potential. Following the knockdown of SGSCs by siRNA against WNT3A, the differentiation potential of SGSCs^3D^ was reduced (Fig. 3e) and the diameters and numbers of acinar-like organoids decreased (Fig. 3f). The phenotypic marker genes of differentiated cells such as acinar cells (*AMY* and *AQP5*), TJ protein (*TJP1*), and intercellular adherence protein (*CDH1*) were also reduced (Additional file 2: Figure S5c), and the corresponding protein translation decreased in the presence of WNT3A siRNA (Fig. 3g). Similarly, upon knockdown by transfection with β-catenin siRNA, differentiation capacity was attenuated, showing the same patterns (Additional file 2: Figure S5d–h).

### 3D-primed SGSCs protect IR-induced salivary damage in a coculture model

**Fig. 4:**
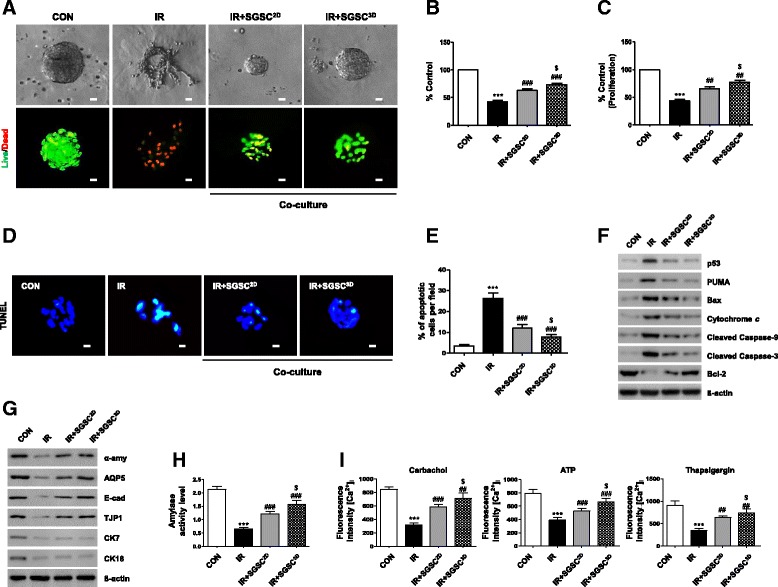
Radioprotective effects of 3D spheroid-derived SGSCs (SGSCs^3D^) in a coculture model. **a**–**c** Effects of SGSCs^3D^ on IR-induced changes in cell morphology, viability, and proliferation of human parotid epithelial cells (hPECs). Scale bars represent 20 μm. Datasets from three independent experiments (*n* = 3). **d** Terminal deoxynucleotidyl transferase (TdT) dUTP nick-end labeling (TUNEL) assays. **e** Comparison of percentages of TUNEL-positive apoptotic cells among groups. Data from three independent experiments presented as mean number of apoptotic cells per field ± SEM (*n* = 3). **f** Western blotting analysis of proapoptotic and antiapoptotic protein levels. **g** Protein levels of salivary acinar markers (α-amylase and AQP5), ductal markers (CK7 and CK18), tight junction protein (TJP1), and adherence protein (E-cadherin) determined by western blotting. **h** Effects of SGSCs^2D^ and SGSCs^3D^ on amylase secretion in hPECs. Amylase activities per wells containing 10^5^ hPECs examined using assay kit. Data from three independent experiments analyzed and presented as mean ± SEM (*n* = 3). **i** Intracellular Ca^2+^ levels measured at baseline and upon stimulation with an agonist. Data presented as mean fluorescence intensity ± SEM. Data from three independent experiments analyzed and presented as mean fluorescent intensity ± SEM (*n* = 3). Two-way ANOVA, Bonferroni’s post hoc test. *Compared to CON in each group; #compared to IR in each group; $compared to SGSCs^2D^ in each group. ***P < 0.001, ^##^*P* < 0.01, ^###^*P* < 0.001, ^$^*P* < 0.05. CON control, IR irradiation, SGSC salivary gland-resident stem cell, SGSCs^2D^ monolayer-cultured SGSCs, [Ca^2+^]_*i*_ intracellular calcium

We next determined whether 3D-primed SGSCs (SGSCs^3D^) have therapeutic beneficial effects on radiation-induced salivary damage. We first used our 3D coculture model in which hPEC spheroids on GFR-Matrigel were irradiated and then cocultured with either SGSCs^2D^ or SGSCs^3D^ cultured on upper inserts, and examined the cellular, structural, and functional damage after coculture. After 2 days of coculture, 3D hPEC spheroids were destroyed in the IR control, but preserved in the coculture with SGSCs (Fig. 4a). A LIVE/DEAD cell assay revealed that this morphological alteration was related to increased cell death after IR, with fewer red-fluorescent dead cells observed after coculture with SGSCs (Fig. 4a). The viability percentage measured using a Trypan blue exclusion assay indicated better viability in the SGSC^3D^ group than in the SGSC^2D^ group (Fig. 4b). Moreover, hPEC spheroids cocultured with SGSCs^3D^ showed a higher rate of proliferation compared with those cocultured with SGSCs^2D^ (Fig. 4c).

A TUNEL assay was performed to examine IR-induced cell death. The amount of fragmented DNA in the hPEC spheroids decreased significantly 2 days after coculture compared with the IR group (Fig. 4d, e). To elucidate the mechanism of IR-induced cell death, we explored changes in the expression of apoptosis-related proteins. The expression of p53 and its proapoptotic target, PUMA, increased in the 3D hPEC spheroids following IR; this change led to the upregulation of proapoptotic proteins including Bax, cytosolic cytochrome c, and cleaved caspases 9 and 3, whereas expression of the antiapoptotic protein Bcl-2 was reduced (Fig. 4f). IR-induced cell death declined during coculture with the SGSCs, and the antiapoptotic effect was more pronounced in the SGSC^3D^ group than in the SGSC^2D^ group (densitometry shown in Additional file 2: Figure S6a). Taken together, our coculture results suggest that 3D priming of the SGSCs enhanced their antiapoptotic effect following IR damage.

Structural maintenance was confirmed by western blotting, in which the levels of expression of salivary epithelial markers (α-amylase, AQP5, E-cadherin, and TJP1) were maintained in the SGSC^3D^-cocultured group compared with the IR and SGSC^2D^-cocultured groups (Fig. 4g, densitometry shown in Additional file 2: Figure S6b). Finally, salivary acinar function was determined from the activity of secreted amylase (Fig. 4h) and intracellular Ca^2+^ concentrations regulating secretion in response to Ca agonists, and exhibited greater preservation when hPEC spheroids were cultured with SGSCs, with more pronounced effects in SGSCs^3D^ than in SGSCs^2D^ (Fig. 4i).

### 3D-primed SGSCs restore IR-induced salivary gland hypofunction in vivo

**Fig. 5:**
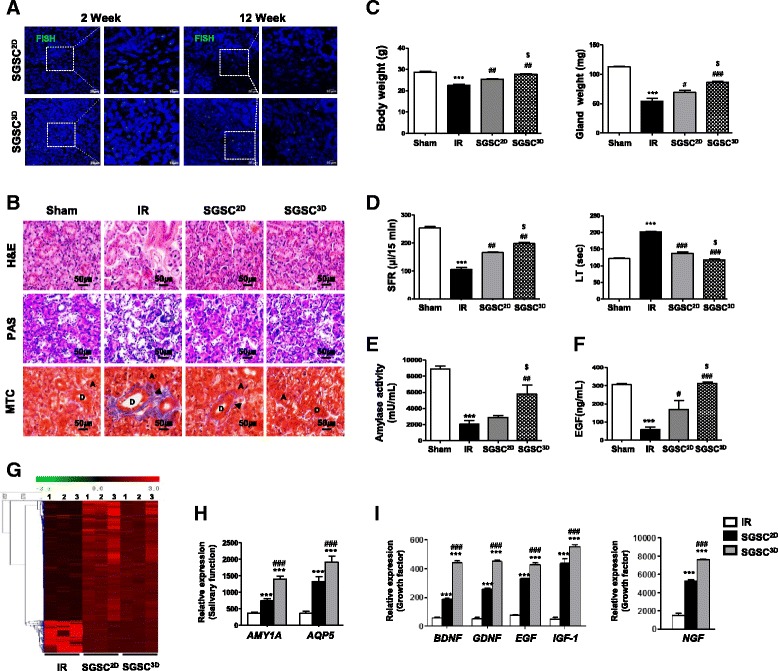
3D-primed salivary gland-resident stem cells (SGSCs) restore IR-induced salivary gland hypofunction in vivo. **a** Fluorescent *in-situ* hybridization (FISH) analysis. **b** Representative histological images of hematoxylin and eosin (H&E), Periodic acid Schiff (PAS), and Masson’s trichrome (MTC) staining from three groups at 12 weeks post IR. Scale bars represent 50 μm. A acinar, D duct. Arrow: fibrosis. **c** Body and gland weights measured at 16 weeks after treatment. **d** Salivary flow rate (SFR) calculated at 16 weeks. Changes in SFR after IR expressed as ratio of post-IR SFR to pre-IR SFR (mean ± SEM). Time to salivation (lag time (LT)) measured and ratios of post-IR LT to pre-IR LT presented. **e** Salivary amylase activity examined using assay kit and fold-changes in activity levels presented. **f** EGF content measured at each time point and average concentrations presented. Data presented as mean ± SEM. One-way ANOVA; Tukey’s post-hoc test. *Compared with Sham group; ^#^compared with IR group; ^$^compared with SGSC^2D^ group. ***P < 0.001, ^#^*P* < 0.05, ^##^*P* < 0.01, ^###^*P* < 0.001, ^$^*P* < 0.05. **g** Differentially regulated genes (> 2-fold) quantified and presented in a heat map indicating antibody reactivity intensity as red (values > 2-fold) and green (values < 2-fold) pixels. **h**, **i** Salivary acinar and growth factor genes in SGSC^3D^ group after whole-genome microarray analysis and comparison with IR and SGSC^2D^ groups. One-way ANOVA; Tukey’s post-hoc test. *Compared with IR group; ^#^compared with SGSC^2D^ group. ***P < 0.001, ^###^*P* < 0.001. SGSCs^2D^ monolayer-cultured SGSCs, SGSCs^3D^ 3D spheroid-derived SGSCs, IR irradiation, EGF epidermal growth factor

We next evaluated the regenerative potential of SGSCs^3D^ relative to SGSCs^2D^ after in-vivo transplantation into mice with IR-induced salivary hypofunction. At 4 weeks after IR, the vehicle (PBS), SGSCs^2D^, or SGSCs^3D^ were transplanted into irradiated salivary glands. Stem cell engraftment was then tracked at 2 and 12 weeks post transplantation. FISH using human Y-chromosome-specific probes in recipient salivary gland tissues revealed that SGSCs^3D^ exhibited greater engraftment in the salivary glands than SGSCs^2D^ at 2 and 12 weeks post transplantation (Fig. 5a and Additional file 2: Figure S7a). At 12 weeks post transplantation, histological examination revealed that PAS-stained, mucin-producing secretory acinar cells were better preserved in SGSC^3D^-treated mice than in SGSC^2D^-treated mice (Fig. 5b and Additional file 2: Figure S7b). MTC staining revealed that fibrosis surrounding ducts or vessels was lower in the SGSC^3D^-treated mice than in the control mice (Fig. 5b and Additional file 2: Figure S7c). The SGSC-treated mice exhibited increased body weight and glandular weight compared with the vehicle (PBS)-injected mice, with better improvement observed in SGSCs^3D^ (Fig. 5c). The salivary flow rate and lag time improved after SGSC treatment, with the best effect observed in SGSCs^3D^ (Fig. 5d). Western blotting showed that more salivary proteins such as amylase and EGF were produced in the SGSC^3D^-treated mice (Additional file 2: Figure S7d). Increases in amylase activity and in the amount of EGF in the saliva were also confirmed (Fig. 5e, f). These salivary function assays indicated that SGSCs^3D^ yielded better results with respect to restoring salivary hypofunction after in-vivo transplantation than SGSCs^2D^. Taken together, 3D spheroid culture in the microwells promoted the stem cell functions of SGSCs, resulting in remodeling of radiation-damaged tissue and restoration of radiation-induced salivary secretory hypofunction.

To determine the in-vivo therapeutic mechanisms, we conducted microarray analysis of in-vivo tissues in the salivary glands of recipient mice (Fig. 5f). We analyzed the differential expression of genes in the vehicle, SGSC^2D^-treated, and SGSC^3D^-treated salivary glands. We found greater expression of salivary epithelial cell markers (*AMY1A* and *AQP5*) in the SGSC^3D^-treated mice than in the SGSC^2D^-treated mice, suggesting the regeneration of salivary epithelial structures (Fig. 5g). The microarray data further revealed that in the SGSC^3D^-treated salivary glands there was higher expression of stem cell-related markers (POU5F1, SOX2, LGR5, THY1) (Additional file 2: Figure S7e) and paracrine factors (BDNF, GDNF, EGF, IGF1, and NGF) (Fig. 5h, i), suggesting that enhanced stemness and paracrine function participate in tissue regeneration and the restoration of damaged function following IR.

## Discussion

In recent years, it has been demonstrated that the 3D culture of stem cells, particularly as spheroids, improves the therapeutic potential of these cells by promoting stemness and/or enhancing paracrine function related to anti-inflammation, anti-apoptosis, or immunomodulation. To improve spheroid formation, we designed an array of microwells formed from a cell-repellent PEG hydrogel micropattern for incorporation onto cell-adherent PCL nanofibrous scaffolds. We then determined whether 3D spheroid culture modulates the stem cell functions of ASCs clonally isolated from salivary glands. We also determined whether such microwell culture can be used to prime SGSCs to improve their therapeutic contribution to restoring radiation-induced salivary hypofunction. Our results demonstrated that SGSCs^3D^ exhibit enhanced stemness in terms of self-renewal, phenotypic marker expression, and multipotent differentiation, and express more paracrine-related factors than SGSCs^2D^. The promoted therapeutic potential was confirmed by coculture experiments and transplantation of SGSCs^3D^ compared with traditional monolayer-cultured SGSCs (SGSC^2D^). This priming process by 3D spheroid culture in microwells activated the Wnt/β-catenin pathway, enabling modulation of SGSC functions and priming SGSCs for a better therapeutic contribution to IR-induced salivary hypofunction.

ASCs have emerged as promising therapeutic candidates for ameliorating or reversing IR-induced salivary hypofunction in many preclinical studies [15–22]. Owing to unmet clinical needs with regard to the prevention or treatment of radiation injuries, including those found in head and neck cancer survivors who suffer from lifelong and critical oral and general side-effects, ASC therapy is extensively investigated to ensure that the optimal cell source, isolation method, and culture conditions are being used. Current approaches for modulating culture conditions to prepare optimized ASCs focus on preconditioning or priming strategies to preactivate stem cell functions prior to transplantation. These include: physiological or chemical preconditioning; genetic engineering; and promoting cell-to-cell or cell-to-ECM interactions using 3D culture [4, 23]. Using different strategies or combined approaches, robust and optimized ASC-based therapeutics have been developed, and their potential has been demonstrated.

The salivary gland is an exocrine gland that produces and excretes saliva into the oral cavity. It consists of a lobular parenchymal compartment of acini and ducts, and surrounding stroma including myoepithelial and neurovascular bundles. The localization of salivary gland-resident stem or progenitor cells remains unclear; we have previously predicted that multipotent stem cells are located close to the ECM of the parenchymal compartment [8]. Because cell-to-ECM interactions play a crucial role in directing cell behavior and properties, we attempted to modulate our single clonal SGSCs by 3D spheroid culture, which recapitulates cell-to-cell and cell-to-ECM interactions in vivo more effectively than traditional 2D monolayer culture.

A variety of culture methods have been developed for 3D culture conditions, including the use of cell-embedding biomaterials, cell sheets, nonadherent dishes, spinner flasks with bioreactors, hanging-drop devices, and microwell arrays [9, 24–30]. We recently fabricated a microwell 3D spheroid culture system comprising a PEG hydrogel wall-micropatterned PCL nanofibrous sheet. The system permits cell-to-ECM interactions by allowing the attachment of cells onto the cell-adherent PCL bottom, and promotes cell-to-cell contact by the use of cell-repellent PEG hydrogel walls between wells. Using this microwell culture, we successfully assembled acinar-like structures when salivary gland epithelial cells were cultured in the appropriate medium. In the present study, we employed this microwell culture system to obtain primed SGSCs for preclinical investigation. In the microwells, the SGSCs readily formed into spheroids by day 7, and exhibited viable and robust SGSC aggregates, which were activated to express the stemness genes *POU5F1* and *NANOG*, and the SGSC markers *LGR5* and *THY1*. Furthermore, the spheroid-derived SGSCs^3D^ exhibited better multipotent differentiation potential when the stem cell medium was changed to differentiation medium.

The optimal culture conditions for priming SGSCs were determined, and we observed that the SGSC spheroids in a 200 × 200 μm^2^ well maintained stem cell properties for 7 days. The expression levels of stem cell-related markers were higher in the SGSCs in 200 μm^2^ wells than in the SGSCs in 100 or 500 μm^2^ wells. Moreover, most spheroidal SGSCs in a 200 μm^2^ well were viable without evidence of hypoxic conditions which may promote stemness of SGSCs^3D^ in the center of the spheroids, possibly because the spheroids were small, which allowed sufficient diffusion of oxygen and nutrients. Furthermore, the spheroid-forming ability during repeated cell aggregation and disaggregation was maintained for up to 10 passages, indicating that SGSCs^3D^ possess highly clonogenic and self-renewing properties, and can be maintained in our 3D microwell culture.

Mesenchymal stem cell (MSC) aggregates have osteogenic or chondrogenic differentiation potential, depending on the culture conditions [24]. Dynamic 3D conditions, topography, or shear stress contribute to the enhanced differentiation of MSCs [31]. In the present study, we determined whether ASCs from salivary glands, which are thought to be multipotent tissue stem cells, could be primed to give rise to salivary epithelial cells under appropriate induction conditions. Our results demonstrated that priming SGSCs by 3D spheroid culture prior to transplant enhanced their therapeutic potential, because the preactivated stem cells were ready to act in vivo with a short time lag after transplantation.

Our microarray data also showed that the improvement in therapeutic potential was not just attributable to the effect of 3D priming culture on the maintenance of stemness and multipotent differentiation potential. DEG analyses revealed that differences in transcripts between SGSCs^2D^ and SGSCs^3D^ promoted paracrine function and enriched transcripts of stemness markers in SGSCs^3D^. There were significant increases in BDNF, GDNF, EGF, HGF, and IGF1 expression in SGSCs^3D^; such proteins are involved in salivary tissue development, remodeling, or the modulation of homeostasis. Although the mechanisms by which such secreted factors act remain unclear, these findings improve our understanding of the therapeutic potential of SGSCs for ameliorating or regenerating radiation-damaged salivary glands.

Wnt is a key molecule in stem cell biology and the growth of embryonic stem cells or ASCs [32]. In particular, Wnt-dependent ASCs from a variety of tissue origins are controlled by Wnt protein excreted from the stem cell niche [33, 34]. Our SGSC clones exhibited LGR5-expressing epitheliomesenchymal features and, as expected, our microarray data revealed that the primed SGSCs^3D^ exhibited enhanced Wnt/β-catenin signaling. When the Wnt signal was inhibited by WNT3A knockdown before 3D priming culture, the Wnt/β-catenin pathway was antagonized, leading to reduced spheroid-forming and differentiation activities. In contrast, Wnt/β-catenin signals were amplified by R-spondin, WNT3A, or β-catenin knockin, further increasing the levels of stemness markers NANOG, SOX2, and POU5F1. Wnt signal amplification in 3D spheroid culture may be attributed to increased cell-to-cell or cell-to-ECM interactions in our microwell culture. Although it remains unclear how these interactions activate Wnt/β-catenin signaling, the results suggest that Wnt/β-catenin signaling involves a priming process that enhances the stem cell properties of salivary ASCs when cultured in microwells to form spheroids.

Under in-vitro coculture of irradiated hPEC spheroids and SGSCs, we verified the antiapoptotic and tissue remodeling effects of 3D-primed SGSCs against radiation damage. Moreover, SGSCs^3D^ exhibited greater enhancement of radioprotective effects than SGSCs^2D^ in terms of radiation-induced growth inhibition, apoptosis, and structural damage with loss of function. This 3D coculture model enables analysis of the therapeutic mechanisms of new radioprotective drugs, and we are currently attempting to identify the relevant key paracrine factors secreted by SGSCs, and evaluate their therapeutic effects on irradiated hPEC spheroids. Furthermore, through in-vivo experiments, we showed that the therapeutic efficacy of SGSCs against IR-induced salivary hypofunction was promoted by 3D priming culture prior to transplantation. The therapeutic mechanism was also attributed to enhanced stem cell functions including enhanced stemness and paracrine activity.

In addition to the efficient modulation of stem cell function, our microwell culture system has other advantages in 3D spheroid formation. Spheroid formation for priming the function of salivary ASCs is controllable and scalable by adjusting the well size in the microwell culture sheet. Moreover, the system enables the mass production of spheroids, which is advantageous for clinical applications. As stem cell niche engineering technology has advanced, stem cell niche factors such as Wnt signals can be engineered for incorporation into scaffolds, beads, or microfluidic devices. In addition to biomaterials for 3D culture, the development of potent enhancers of stem cell functions, including small molecules, biologics, or genes, may greatly facilitate stem cell engineering or priming processes prior to clinical use. Although we showed that this 3D priming strategy of human SGSCs is attractive for future clinical applications, further studies are necessary to determine the optimal timing and delivery of SGSCs^3D^, as well as to identify the key trophic factors released from SGSCs^3D^, and the related molecular pathway mediating the radioprotective effects for future clinical applications.

## Conclusions

In summary, we established a 3D priming culture system for SGSCs comprising microwell sheets for use prior to therapy to promote stem cell functions. Wnt signaling is activated by 3D spheroid formation in microwells, enabling recapitulation of the 3D microenvironment for SGSCs and driving enhanced stemness and paracrine function. This 3D priming culture for use prior to the transplantation of SGSCs is beneficial for therapy aimed at treating IR-damaged salivary hypofunction.

## Additional files

Additional file 1: **Table S1.** Presenting primers used for RT-PCR. (PDF 975 kb)

Additional file 2: **Figures S1–S7.** Showing supplementary results. (PDF 2615 kb)

## Abbreviations

3D: Three-dimensional
ASC: Adult stem cell
DAPI: 4′,6-Diamidino-2-phenylindole dihydrochloride
DMEM: Dulbecco’s Modified Eagle Medium
ECM: Extracellular matrix
EGF: Epidermal growth factor
FBS: Fetal bovine serum
HGF: Hepatocyte growth factor
IGF: Insulin growth factor
IR: Irradiation
PCL: Polycaprolactone
PEG-DA: Poly(ethylene glycol)-diacrylate
qRT-PCR: Quantitative reverse transcription-polymerase chain reaction
SGSC: Salivary gland-resident stem cell
SGSCs^2D^: Monolayer-cultured SGSCs
SGSCs^3D^: 3D spheroid-derived SGSCs
TJP: Tight junction protein
VEGF: Vascular endothelial growth factor
