# Supplementary material for: Stem cell properties of human clonal salivary gland stem cells are enhanced by three-dimensional priming culture in nanofibrous microwells
Source: Stem Cell Res Ther. 2018 Mar 22;9:74. doi: 10.1186/s13287-018-0829-x (PMC5863805; doi:10.1186/s13287-018-0829-x)
Supplement: Supplementary file 1 — Table S1. Presenting primers used for RT-PCR. (PDF 975 kb) [file 13287_2018_829_MOESM1_ESM.pdf]

Stem Cell Properties of Human Clonal Salivary Gland Stem Cells Are Enhanced by Three-dimensional Priming Culture in Nanofibrous Microwells

Running Title: Three-dimensional Priming Culture of Salivary Gland Stem Cells

Hyun-Soo Shin[1], Songyi Lee[1], Hye Jin Hong[2], Won-Gun Koh[2], Young Chang Lim[3], Jae-Yol Lim[1,*]

[1]Department of Otorhinolaryngology, Yonsei University College of Medicine;

[2]Department of Chemical and Biomolecular Engineering, Yonsei University;

[3]Department of Otorhinolaryngology, Head and Neck Surgery, Konkuk University School of Medicine; Seoul, Republic of Korea

***Corresponding author**: Jae-Yol Lim, MD, PhD, Department of Otorhinolaryngology, Gangnam Severance Hospital, Yonsei University College of Medicine, 211 Eonju-ro, Gangnam-gu, Seoul 06273, Republic of Korea

E-mail: jylimmd@yuhs.ac Tel: 82-2-2019-3460, Fax: 82-2-3463-4750

Table S1. Primers used for RT-PCR.

Gene and symbol		Primer sequences (5’-3’)
AMY1A	F	AATTGATCTGGGTGGTGAGC
	R	CTTATTTGGCGCCATCGATG
AQP5	F	ACTGGGTTTTCTGGGTAGGG
	R	GTGGTCAGCTCCATGGTCTT
CDH1	F	CGCATTGCCACATACACTCT
	R	TTGGCTGAGGATGGTGTA AG
TJP	F	TTTGGCCGAGGGATAGAAGT
	R	TATTGCCATCTCTTGCTGCC
POU5F1	F	AAGCGATCAAGCAGCGACTA
	R	GAAGTGAGGGCTCCCATAGC
NANOG	F	CAGAAAAACAACTGGCCGAA
	R	GGCCTGATTGTTCCAGGATT
LGR5	F	AGGATCTGGTGAGCCTGAGAA
	R	CATAAGTGATGCTGGAGCTGGTAA
ITGB1	F	ATGGGCAGAGCAATGGTGGCCA
	R	AGAGTGAGACC ACGAAGAGACT
THY1	F	GACAGCCTGAGAGGGTCTTG
	R	CCCAGTGAAGATGCAGGTTT
SOX2	F	CAACATGATGGAGACGGAGC
	R	GTGCATCTTGGGGTTCTCCT
CTNNB1	F	GTGTGGAGACAGGGGCTTTT
	R	GGTGCGCAATACATCTCCAG
AXIN2	F	CAAGGCCAGCACATAGGAGA
	R	GCAACGCGAGTCTGTGTTTT
WNT3A	F	GGCCCCACTCGGATACTTCT
	R	AGGGAGGAATACTGTGGCCC
WNT9A	F	AAGGTGATCAAGGCTGGGGT
	R	CTCATGGAAAGGCGCCAACT
ACTB	F	AGCTGTGCTATGTTGCCCTG
	R	AGGAAGCAAGGCTGGAAGAG
GAPDH	F	ATGGGGAAGGTGAAGGTCG
	R	TAAAAGCAGCCCTGGTGACC

LPL	F	TACAGGGCGGCCACAAGTTTT
	R	ATGGAGAGCAAAGCCCTGCTC
FABP4	F	CATCAGTGTGAATGGGGATG
	R	GTGGAAGTGACGCCTTTCAT
PPARG2	F	GACCACTCCCACTCCTTTGA
	R	CGACATTCAATTGCCATGAG
RUNX2	F	TATGAAAAACCAAGTAGCAAGGTTC
	R	GTAATCTGACTCTGTCCTTGTGGAT
BGLAP	F	GTGCAGAGTCCAGCAAAGGT
	R	CTAGCCAACTCGTCACAGTC
ACAN	F	GCTACACCCTAAAGCCACTGCT
	R	CGTAGTGCTCCTCATGGTCATC
COL2	F	TTTCCCACCTCAAGATGGTC
	R	TCACCTGGTTTTCCACCTTC
COL10	F	GCCCAAGAGGTGCCCCTGGAATAC
	R	CCTGAGAAAGAGGAGTGGACATAC
