# Supplementary material for: Stem cell properties of human clonal salivary gland stem cells are enhanced by three-dimensional priming culture in nanofibrous microwells
Source: Stem Cell Res Ther. 2018 Mar 22;9:74. doi: 10.1186/s13287-018-0829-x (PMC5863805; doi:10.1186/s13287-018-0829-x)
Supplement: Supplementary file 2 — Figures S1–S7. Showing supplementary results. (PDF 2615 kb) [file 13287_2018_829_MOESM2_ESM.pdf]

Stem Cell Properties of Human Clonal Salivary Gland Stem Cells Are Enhanced by Three-dimensional Priming Culture in Nanofibrous Microwells

Running Title: Three-dimensional Priming Culture of Salivary Gland Stem Cells

Hyun-Soo Shin[1], Songyi Lee[1], Hye Jin Hong[2], Won-Gun Koh[2], Young Chang Lim[3], Jae-Yol Lim[1,*]

[1]Department of Otorhinolaryngology, Yonsei University College of Medicine;

[2]Department of Chemical and Biomolecular Engineering, Yonsei University;

[3]Department of Otorhinolaryngology, Head and Neck Surgery, Konkuk University School of Medicine; Seoul, Republic of Korea

***Corresponding author**: Jae-Yol Lim, MD, PhD, Department of Otorhinolaryngology, Gangnam Severance Hospital, Yonsei University College of Medicine, 211 Eonju-ro, Gangnam-gu, Seoul 06273, Republic of Korea

E-mail: jylimmd@yuhs.ac Tel: 82-2-2019-3460, Fax: 82-2-3463-4750

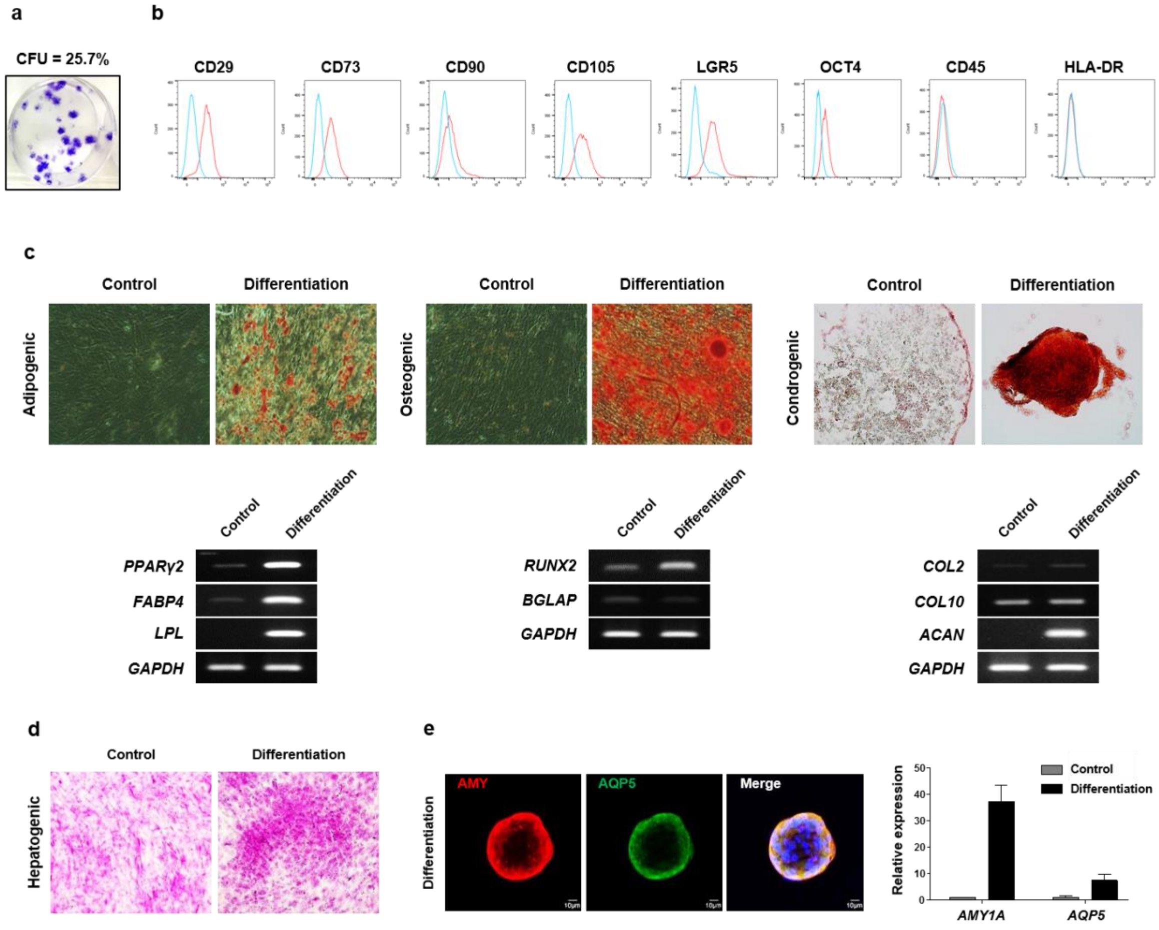

Figure S1. (**a**) The isolated SGSCs showed colony-forming unit activity of 25.7%, which indicated that the isolated clonal SGSCs possess the stem cell property of self-renewal. (b) Flow cytometric analysis was conducted to monitor the expression of the markers of SGSCs. SGSCs expressed mesenchymal markers (CD29, CD73, CD90, and CD105), an epithelial stem cell marker (LGR5), and a pluripotent marker (POU5F1). (c) Each clonal population was appropriately induced to differentiate into three mesenchymal cell types. At the end of each differentiation, the cells were stained with oil red O, alizarin red S, and safranin O to evaluate adipogenic, osteogenic, and chondrogenic differentiation, respectively. The magnification is 100× and 200× in the osteogenic/chondrogenic differentiation and adipogenic differentiation panels, respectively. In addition to specific cytochemical staining, the expression of molecular markers for each differentiation was also analyzed by RT-PCR. (d) To assess the multiple epithelial differentiation potential of the SGSCs, each clonal population was

subject to *in vitro* hepatogenic differentiation. The differentiation was evaluated by PAS staining. (e) SGSCs were allowed to form salisphere-like cell structure followed by salivary epithelial cell differentiation. Salivary epithelial differentiation was evaluated by immunofluorescence staining for AQP5 and α-amylase. Scale bars = 20 μm. The expression of salivary epithelial differentiation-associated molecular markers including *AMY1A* and *AQP5* were examined by semi-quantitative RT-PCR.

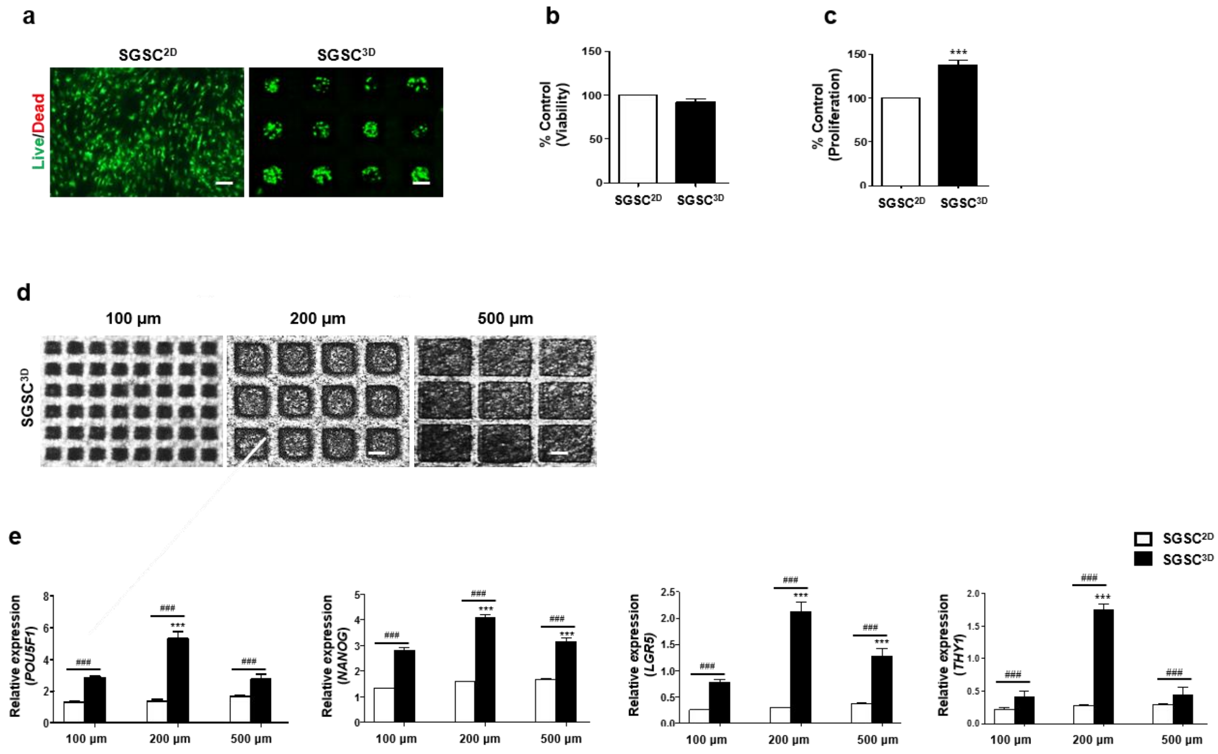

Figure S2. 3D spheroid formation from single clonal salivary gland-resident stem cells (SGSCs). (a) Representative LIVE/DEAD fluorescence image of SGSC spheroids at 5 days after culture. Scale bars represent 100 μm. (b) Percentage cell viability (viable cell count/total cell count) was measured at 5 days using the Trypan blue dye exclusion technique (c) Cell proliferation of SGSCs^{2D} and SGSCs^{3D} was examined at 5 days. Data from three independent experiments were analyzed and are presented as the mean ± SEM (n = 3). One-way ANOVA; Tukey's post hoc test: *, compared with SGSC^{2D} (d) Light microscope images of SGSCs at 5 days after culture in microwells of various sizes. Scale bars represent 100 μm. (e) Transcription levels of pluripotent markers (*POU5F1* and *NANOG*), salivary stem cell markers (*LGR5* and *THY1*) confirmed by quantitative polymerase chain reaction (qPCR) at 5 days after culture in microwells of various sizes. Data from three independent experiments were analyzed and presented as the mean ± SEM (n = 3). Two-way ANOVA, Bonferroni's post

hoc test. *, compared with 100 μm; #, compared with monolayer-cultured SGSCs (SGSCs2D) in each group. ***$P < 0.001$, ###$P < 0.001$.

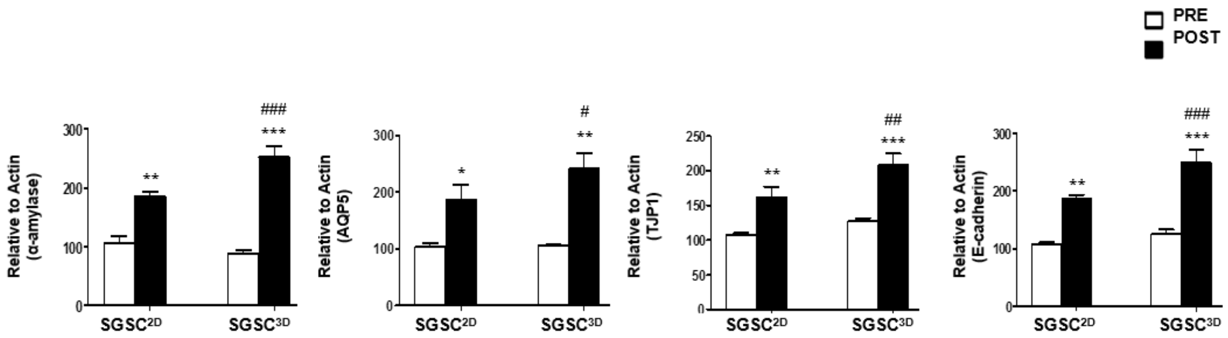

Figure S3. Salivary phenotypic marker and stem cell marker changes after induction of differentiation. (Densitometry from Fig 1F) Protein levels of SG acinar cell markers (α-amylase and AQP5), tight junction protein (TJP1), intercellular adherence protein (E-cadherin) were determined by western blotting in SGSCs^{2D} and SGSCs^{3D} cultures after differentiation. Data from three independent experiments were analyzed and densitometric data are presented as the mean ± SEM (n = 3). Two-way ANOVA, Bonferroni's post hoc test. *, comparison between PRE and POST; #, compared with the SGSCs^{2D} group. *P < 0.05, **P < 0.01, ***P < 0.001, #P < 0.05, ##P < 0.01, ###P < 0.001.

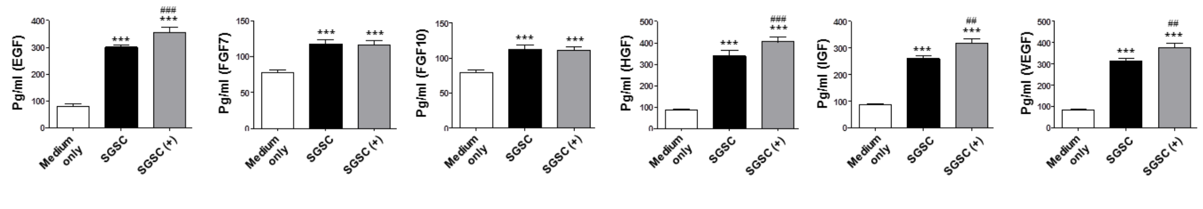

Figure S4. Concentrations of growth factors released by SGSCs were measured under 2D and 3D culture conditions. One-way ANOVA, Tukey's post-hoc test: *compared to the medium only; #compared to group SGSCs^{2D}. ***P < 0.001, ##P < 0.01, ###P < 0.001

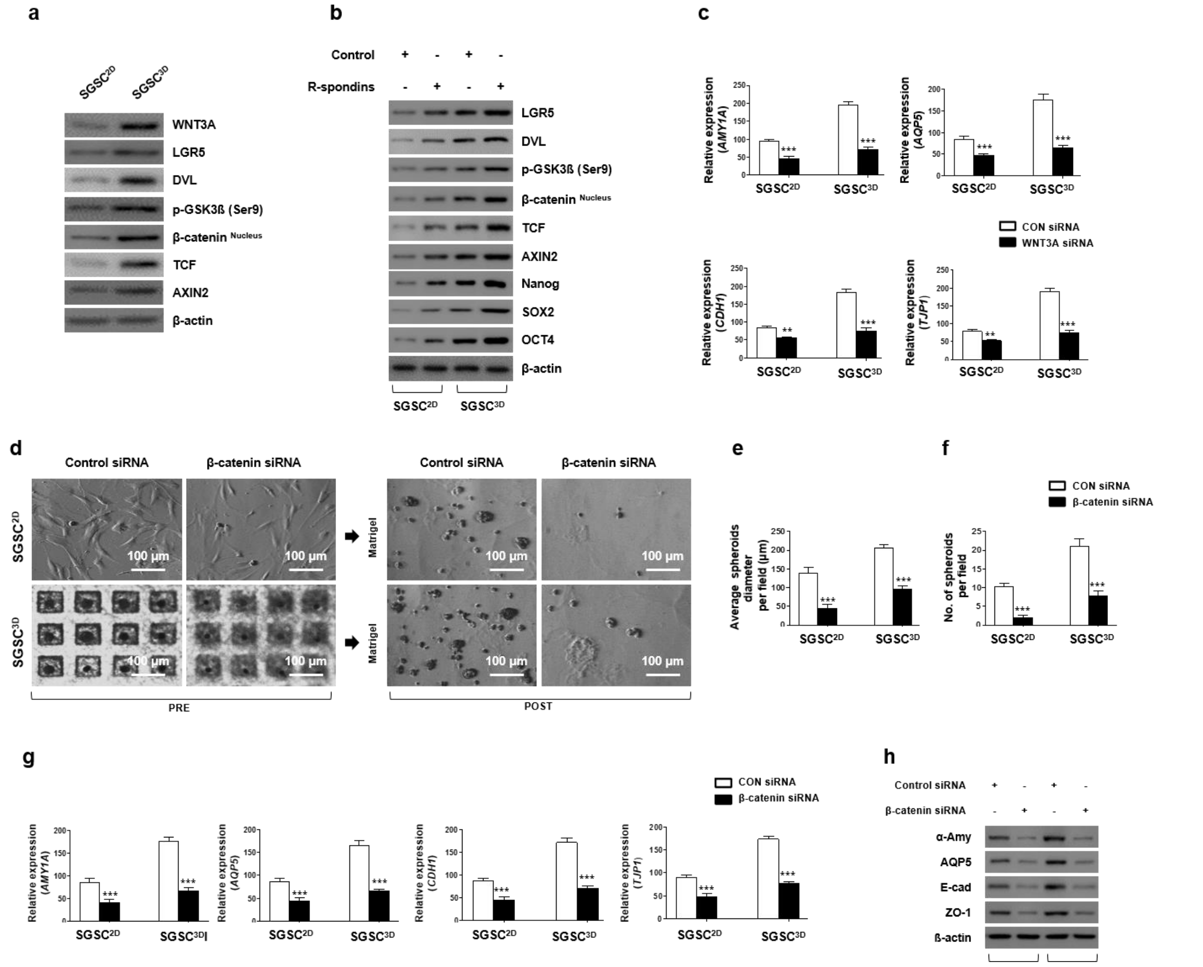

Figure S5. (a) 3D spheroid-derived salivary gland-resident stem cell ($SGSC^{3D}$) culture induced WNT-β-catenin-related proteins (WNT3A, LGR5, DVL, GSK3β, nucleus β-catenin, TCF, and AXIN2) and stem cell-related proteins (NANOG, SOX2, and POU5F1). (b) R-spondin upregulated WNT3A-β-catenin-related and stem cell-related proteins. (c) mRNA levels of SG acinar cell markers (α-amylase (*AMY1A*) and AQP5 (*AQP5*)), tight junction proteins (ZO-1 (*TJP1*)), and intercellular adherence protein (E-cadherin (*CDH1*)) were determined by real-time polymerase chain reaction (PCR). Data from three independent experiments were analyzed and are presented as the mean ± SEM (n = 9). One-way ANOVA; Tukey's post hoc test: *, compared with WNT3A siRNA, $^{**}P < 0.01$, $^{***}P < 0.001$. (d) β-catenin siRNA caused morphological changes in the SGSCs. Scale bars represent 100 μm. (e) The diameter of the spheroids was measured after addition of the β-catenin inhibitor, and values

were normalized to the total number of spheroids. Data from five independent experiments were analyzed and are presented as the mean ± SEM (n = 5). One-way ANOVA; Tukey's post hoc test: *, compared with β-catenin siRNA, $^{***}P < 0.001$. (f) Differentiation capacity was determined by measuring the average number of spheroids per plate after plating the same number of cells. Data from five independent experiments were analyzed and are presented as the mean ± SEM (n = 5). One-way ANOVA; Tukey's post hoc test: *, compared with β-catenin siRNA, $^{***}P < 0.001$. (g and h) mRNA and protein levels of SG acinar cell markers (α-amylase (*AMY1A*) and AQP5 (*AQP5*)), tight junction protein (ZO-1 (*TJP*)), and intercellular adherence protein (E-cadherin (*CDH1*)) were determined by real-time polymerase chain reaction (PCR) and western blotting in spheroids after WNT3A inhibition. Data from three independent experiments were analyzed and are presented as the mean ± SEM. One-way ANOVA; Tukey's post hoc test: *, compared with β-catenin siRNA, $^{***}P < 0.001$.

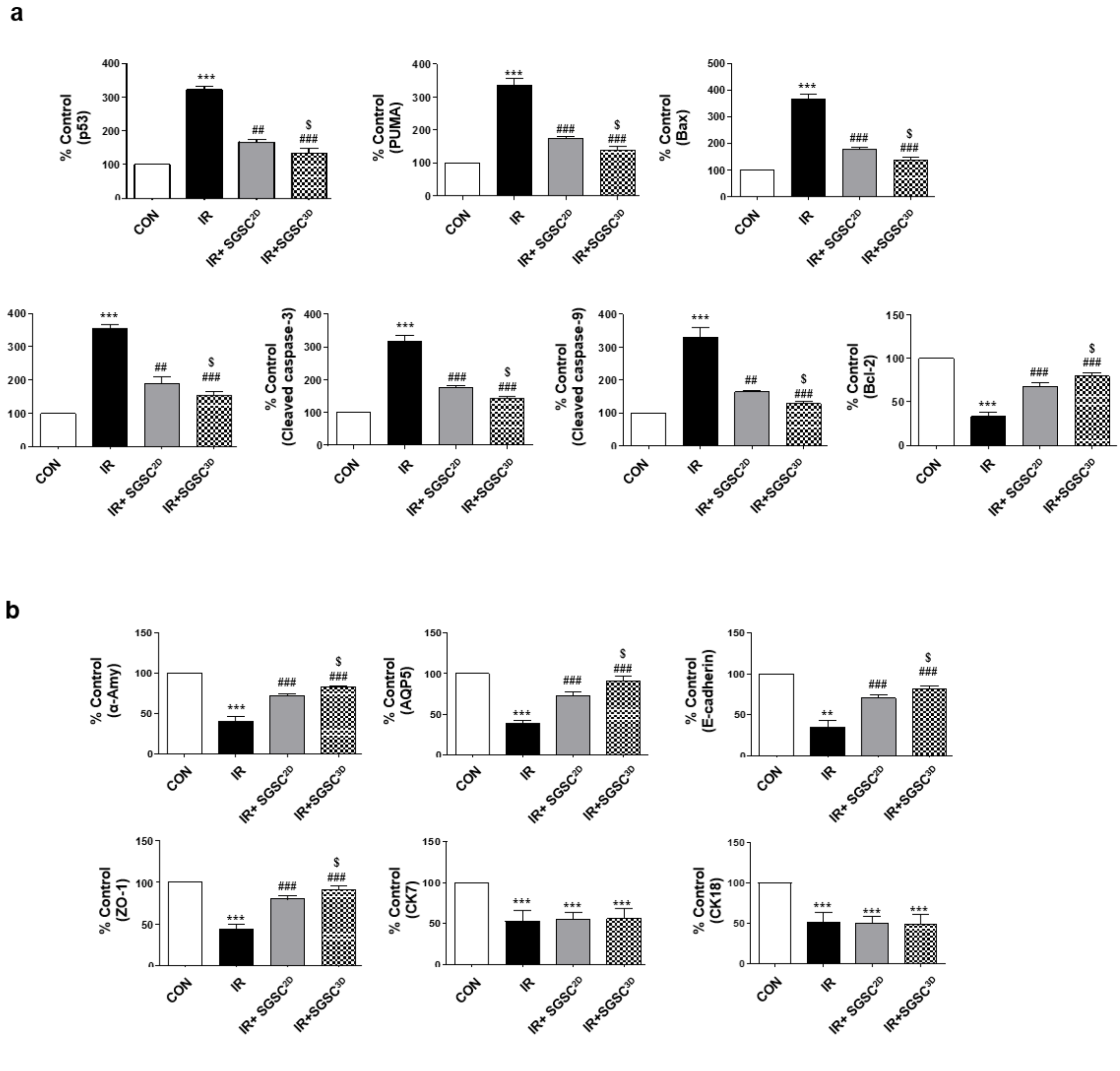

Figure S6. (a) (Densitometry from Fig 6F) Western blot analysis of protein levels of p53, PUMA, Bax, cytochrome c, cleaved caspase 9, cleaved caspase 3, and Bcl-2. Data from three independent experiments were analyzed and presented as the mean ± SEM (n = 3). One-way ANOVA, Tukey's *post-hoc* test: *compared to group CON; $^{\#}$compared to group IR; $^{\$}$compared to group SGSC^{2D}. $^{***}P < 0.001$, $^{\#\#}P < 0.01$, $^{\#\#\#}P < 0.001$, $^{\$}P < 0.05$. (b) (Densitometry from Fig 6G) Western blot analysis of protein levels of salivary acinar markers (α-amylase and AQP5), tight junction protein (TJP1), adherence proteins (E-cadherin), and ductal markers

(CK7 and CK18). Data from three independent experiments were analyzed and presented as the mean ± SEM (n = 3). One-way ANOVA, Tukey's *post-hoc* test: *compared to group CON; #compared to group IR; $compared to group SGSC^{2D}. ***$P < 0.001$, ###$P < 0.001$, $P < 0.05$.

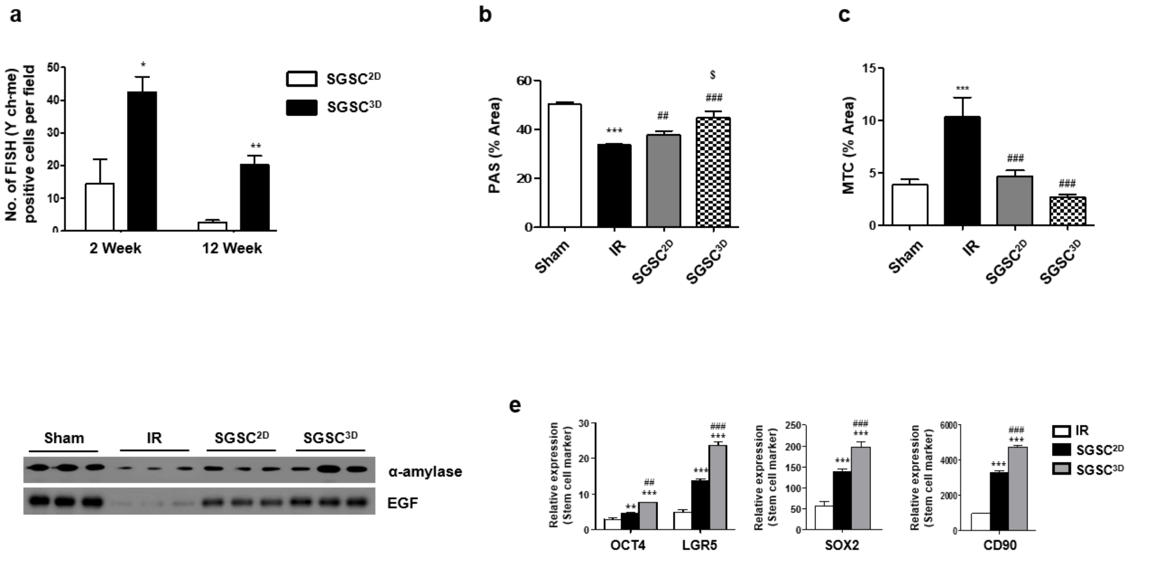

Figure S7. (a) The number of FISH-positive cells at 12 weeks after treatment.

Results are presented as the mean ± SEM One-way ANOVA, Tukey's post hoc test. *, compared to monolayer, $^{*}P < 0.05$, $^{**}P < 0.01$. (b and c) Densities after PAS staining were measured using software to calculate pixels of purple-stained mucin-containing areas. MTC staining revealed that the IR+SGSCs group had less periductal and perivascular fibrosis than the IR group. One-way ANOVA, Tukey's *post-hoc* test: *compared to group CON; $^{\#}$compared to group IR; $^{\$}$compared to group IR+ monolayer. $^{***}P < 0.001$, $^{\#\#}P < 0.01$, $^{\#\#\#}P < 0.001$, $^{\$}P < 0.05$. (d) Western blotting of amylase and EGF in saliva at 16 weeks post-IR. (e) Salivary acinar and growth factor genes in the SGSCs^{3D} group after whole-genome microarray analysis and comparison with the IR and SGSCs^{2D} groups. One-way ANOVA; Tukey's post-hoc test: *, compared with the IR group; $^{\#}$, compared with the SGSCs^{2D} group. $^{***}P < 0.001$, $^{\#\#\#}P < 0.001$.
